# How perceived eWOM in visual form influences online purchase intention on social media: A research based on the SOR theory

**DOI:** 10.1371/journal.pone.0328093

**Published:** 2025-07-10

**Authors:** Chi Thanh Bui, Thi Thuy An Ngo, Huynh Khanh Long Chau, Nguyen Phuc Nguyen Tran

**Affiliations:** 1 Department of Business, FPT University, Can Tho, Vietnam; 2 Department of Soft Skills, FPT University, Can Tho, Vietnam; Al-Ahliyya Amman University, JORDAN

## Abstract

In today’s digital landscape, visual content plays a crucial role in shaping consumer behavior. This study explores how visual electronic word-of-mouth (eWOM) on social media influences online purchase intention, applying the Stimulus-Organism-Response (SOR) framework. Using Partial Least Squares Structural Equation Modeling (PLS-SEM) to analyze data from 335 social media users, this study examines the effects of visual eWOM’s quality, quantity, and credibility on consumer perceptions, attitudes, and ultimately their purchase intentions. Our findings reveal that the quality and credibility of visual eWOM significantly enhance perceived information usefulness and its adoption by consumers. Information quantity, however, primarily influences attitudes towards the information, but does not directly drive its adoption. Contrary to expectations, information usefulness alone cannot predict purchase intention. Instead, information adoption emerges as a key mediator, indicating that consumers must actively engage with and internalize visual content for it to impact their buying behavior. This underscores that the effectiveness of visual eWOM is not solely based on its characteristics but depends on consumers’ active engagement and processing. These insights highlight the need for content that is not only visually appealing but also credible and engaging to facilitate information adoption and drive purchase intentions. This study enhances the understanding of visual eWOM’s impact on online purchasing and provides valuable insights for marketers aiming to optimize digital engagement strategies.

## 1. Introduction

In the contemporary digital landscape, saturated with visual content, the influence of visual communication is profound [1]. Recent statistics highlight its profound influence, with an estimated 14 billion images shared daily across social media platforms [2]. Facebook videos alone garner over 4 billion views per day, while TikTok users spend an average of 22.9 hours per month engaging with video content [3]. The dominance of visual platforms is further evident in their vast user bases, with Facebook boasting 3.04 billion users, YouTube 2.5 billion, Instagram 2 billion, and TikTok 1.5 billion [4]. These figures underscore the immense scale and impact of visual communication, emphasizing the need for brands and individuals to strategically leverage visuals to maximize engagement and influence in the digital space. The explosive popularity of visual platforms such as YouTube, Instagram, Pinterest, and TikTok exemplifies the power of visual communication in capturing user attention and shaping perceptions [3,4]. For instance, Instagram and Pinterest prioritize aesthetics, often leading consumers to value visual appeal over product functionality [5]. Conversely, TikTok’s short-form video format tends to emphasize entertainment and emotional response rather than in-depth product analysis [6]. YouTube, with its global reach product review, and unboxing genres, creates a unique context where social influence and emotional connection with the reviewer can significantly impact purchase decisions [7]. This shift toward visual content is not merely a trend but a fundamental transformation in consumer behavior, reinforcing the growing importance of studying visual electronic word-of-mouth (eWOM). As social media platforms continuously refine their algorithms to prioritize engaging and relevant content, understanding how these changes influence the effectiveness of visual eWOM has become increasingly critical. This research addresses this evolving digital landscape, offering timely insights into how visual eWOM shapes consumer decision-making in an era where online content consumption is driven by algorithmic curation.

While the significance of eWOM is widely acknowledged in consumer research, the literature predominantly focuses on textual forms of eWOM. Online reviews and ratings provide detailed information and personal experiences, aiding potential buyers in making informed choices [8]. Additionally, eWOM can enhance brand awareness and trust [9], with cultural nuances influencing its expression. For example, eWOM in Western cultures often appears more direct and consumer-centric [10]. The emotional dimension of eWOM is also significant, influencing consumer engagement and decision-making [11]. However, this focus on textual eWOM neglects the rising prevalence and impact of visual content in the digital landscape, where images and videos increasingly dominate consumer communication and experience sharing. In fact, the speed at which new visual trends and formats emerge requires a continuous reassessment of their impact on consumer behavior.

In today’s digital era, visual eWOM holds significant importance for several reasons. Visual content is inherently more engaging and memorable than text, capturing attention more quickly and conveying complex information succinctly, thereby enhancing persuasive communication [12]. Visuals, particularly user-generated content such as photos and videos, are often perceived as more authentic and trustworthy, significantly influencing consumer behavior and decision-making processes [13]. Social media platforms amplify the reach of visual eWOM through likes, shares, and comments, with visual content that garners high engagement benefiting from social proof, thus driving consumer actions and brand perceptions. Brands recognize the power of visual eWOM and actively encourage customers to share their experiences visually, integrating user-generated visual content into their marketing strategies to build brand loyalty, enhance customer engagement, and foster a sense of community. Additionally, visual eWOM empowers consumers by providing a platform to voice their opinions and share their experiences with a broad audience, increasing consumer satisfaction and loyalty as individuals feel heard and valued by their peers and brands [14].

Previous research on visual eWOM has highlighted its significant impact on consumer behavior and decision-making. Visual eWOM has been found to enhance the credibility and perceived usefulness of online reviews, leading to increased trust and purchase intentions among consumers [15]. Factors influencing visual eWOM, particularly in the context of restaurant experiences, include the aesthetic appeal of the food presentation, the ambiance of the restaurant, and social influences, all of which contribute to enhancing the restaurant’s reputation [16]. The importance of visual eWOM over traditional text-based reviews has been emphasized, with visual content providing more immediate and impactful information that aids consumers in making informed decisions and enhancing engagement and trust [17]. This study explored how the vividness and interactivity of visual eWOM on platforms like Instagram and YouTube enhance emotional engagement and trust; and revealed that visually rich eWOM content significantly increases consumers’ intention to purchase by effectively engaging their emotions and building trust. Moreover, in the fashion industry, visual content has been found to positively impact purchasing behavior by providing social proof and enhancing perceived value [18]. These findings underscore the importance of visual elements in eWOM, demonstrating their role in shaping consumer perceptions and behaviors.

To address this critical gap in the literature, this study investigates the complex relationship between visual eWOM on social media and consumer purchase intention. Using the Stimulus-Organism-Response (SOR) theory as a framework [19], the study aims to unravel the mechanisms through which the quality, quantity, and credibility of visual eWOM on different platforms evoke cognitive and affective responses in consumers, ultimately shaping their purchase intentions. This research further examines the mediating role of information adoption, the process of internalizing and utilizing visual information in the relationship between perceived visual eWOM stimuli, perceived information usefulness, customer attitudes toward the information, and purchase intention. Given ever-changing algorithms and user preferences on social media, understanding this process is not a one-time endeavor, but a continuous need for researchers and marketers. The study assesses how adopted information shapes consumer attitudes and drives purchase intentions. By addressing these research questions, this study aims to advance the theoretical understanding of visual eWOM on social media platforms and provide practical insights for marketers and consumers alike. The findings will contribute to leveraging the power of visual storytelling and eWOM effectively and ethically while empowering consumers to navigate the landscape of product endorsements and make informed purchase choices.

## 2. Literature review and research framework

### 2.1. Visual eWOM

The increasing dominance of visual content in the digital landscape has shifted the focus of eWOM research from traditional textual reviews and ratings to visual forms [20]. Visual eWOM, which includes images, videos, and other visual content shared by consumers online, has emerged as a powerful influencer of consumer behavior, particularly in shaping purchase intentions. Unlike textual information, visual content is inherently more engaging and memorable. It captures attention more swiftly, conveys complex information more effectively, and elicits stronger emotional responses, enhancing its persuasive impact [21]. User-generated visual content is especially impactful, often perceived as more authentic and trustworthy than content generated by brands, making it a critical factor in consumer decision-making [22].

The persuasive power of visual eWOM has been documented in the literature, demonstrating its superiority over traditional text-based reviews in influencing consumer behavior. Filieri et al. [17] emphasize that visual content provides more immediate and impactful information, aiding consumers in making informed decisions. The vividness and clarity inherent in visual eWOM, such as images and videos, make it a potent tool for conveying key product attributes and benefits. This sensory richness allows consumers to quickly grasp essential features and quality indicators, facilitating quicker and more confident purchase decisions. Zhang et al. [23] further explores the role of visual eWOM in shaping consumer attitudes. They highlight that visual content enhances perceived informativeness and entertainment value, crucial factors in engaging consumers. Visually appealing eWOM content often provides detailed product information, which can be more easily processed and retained compared to text. This dual function of informing and entertaining not only captures consumer attention but also leads to higher levels of information adoption. When consumers find visual eWOM both informative and engaging, they are more likely to perceive the content as credible and valuable, increasing the likelihood of adopting the information. Liu et al. [24] also supports the notion that visually rich eWOM enhances consumer engagement. They argue that well-designed visual content, such as high-quality images or engaging videos, can significantly elevate purchase intention. This effect is attributed to the ability of visual content to create a more immersive and interactive experience, allowing consumers to visualize the product in real-life scenarios. The immersive nature of visual eWOM can evoke strong emotional responses, which play a crucial role in decision-making processes.

Visual eWOM takes multiple forms, including static images, short videos, and long-form videos, each serving a unique purpose in shaping consumer perceptions [13]. High-quality images provide a quick and digestible representation of products or experiences, enhancing consumer confidence and purchase intention [10]. Platforms like Instagram and Pinterest have become central to visual eWOM due to their image-centric nature. Short videos, commonly featured on TikTok, Instagram Reels, and YouTube Shorts, deliver dynamic, easily digestible content that quickly captures attention. These videos effectively showcase product features and user testimonials, fostering trust by offering realistic portrayals of products in use [14]. Research indicates that these short videos foster higher trust levels by offering realistic portrayals of products in action [6]. Platforms like Instagram integrate both images and short videos to create aspirational and emotionally engaging eWOM, leveraging visual storytelling and influencer marketing to shape brand perception and influence purchasing behavior [13]. Long-form videos, such as detailed product reviews, unboxing videos, and tutorials, provide comprehensive demonstrations and comparisons, enabling consumers to make more informed decisions [7]. Research suggests that visually rich eWOM enhances consumer engagement by creating immersive experiences that evoke strong emotional responses, ultimately impacting decision-making [24]. The ability of visual eWOM to seamlessly merge entertainment with detailed product information differentiates it from traditional eWOM, making it a more influential tool in shaping consumer behavior in the evolving digital marketplace.

The unique influence of visual eWOM has been studied across various industries. In the context of restaurant experiences, visual eWOM was found to have significant impacts on consumer perceptions and behaviors. Abdullah et al. [16] revealed that the aesthetic appeal of food presentation, the ambiance of the restaurant, and social influences depicted in visual content play crucial roles in shaping a restaurant’s reputation and consumer patronage. This suggests that consumers are influenced not only by the quality of the food but also by the overall visual experience shared by other patrons. In the fashion industry, visual eWOM has been shown to enhance purchasing behavior by providing social proof and increasing perceived value. Hussain et al. [25] demonstrated that images and videos showcasing clothing and accessories can significantly boost consumer purchase intentions. This is particularly relevant for experience goods, where visual content can provide a more tangible sense of the product’s quality and appeal. Similarly, Poirier et al. [26] highlighted the importance of visual eWOM in influencing purchase decisions, noting that product images often have a stronger impact on consumer intentions than textual descriptions.

Collectively, these studies underline the importance of visual eWOM in the digital marketing landscape. Visual content’s ability to provide immediate, clear, and engaging information makes it a highly effective medium for influencing consumer attitudes and behaviors.

### 2.2. Stimulus-organism-response (SOR) theory

The Stimulus-Organism-Response (SOR) model, proposed by Mehrabian and Russell [19], is a foundational framework for understanding how environmental stimuli impact individual behavior through internal states. Initially developed in psychology, this model has been widely applied in marketing and information systems to elucidate the process by which external stimuli (S) influence an organism’s internal states (O) such as emotions, perceptions, and cognitions which subsequently lead to specific responses (R) like purchasing decisions or social interactions [27,28]. Over time, research has expanded the “organism” component to encompass a broader array of psychological and cognitive processes, including attitudes, beliefs, and evaluations [29].

In the context of visual eWOM, the SOR model provides a robust framework for understanding how visual stimuli in online environments influence consumer behavior. These visual elements affect individuals’ internal states, such as emotions, trust, and perceptions of usefulness, which then impact behavioral responses like information sharing, purchase intentions, and brand loyalty [30]. Research by Debataraja et al. [31] applies the SOR framework to examine how different eWOM characteristics influence purchase intentions. Their findings reveal that customer reviews significantly impact consumers’ purchase intentions (the “response”) due to their perceived authenticity and diverse perspectives [32]. This demonstrates how stimuli (eWOM characteristics) influence the organism (consumer perceptions) and elicit a response (purchase intentions).

The role of visuals within the SOR model, suggests that visual stimuli in eWOM affect consumers’ perceptions of credibility, value, and interest [33]. Their research indicated that images and videos enhance the perceived believability of reviews by showcasing product conditions and practical use cases, while compelling visuals increase the perceived value of eWOM by illustrating product functionality and benefits. Zhang and Shah [34] delved into the psychological mechanisms underpinning the SOR model in the context of eWOM, highlighting the importance of perceived enjoyment and emotional arousal as mediators between visual stimuli and behavioral responses [35]. Their findings suggest that visually appealing eWOM can evoke positive emotions and enjoyment, enhancing the likelihood of information sharing and purchase intentions.

#### 2.2.1. The Stimuli (S).

In the SOR model, the “stimulus” refers to external factors that initiate cognitive and affective processes in consumers [19]. Within the realm of visual eWOM, these stimuli are characterized by information quality, quantity, and credibility. High-quality information is notably influential, as it is perceived as more trustworthy and persuasive, thereby significantly shaping consumer attitudes and behaviors [36]. Accurate and relevant eWOM enhances its credibility, thereby affecting consumers’ perceptions of the product or service being reviewed [37]. Comprehensive eWOM, which provides detailed and thorough information can reduce consumer uncertainty and perceived risk, leading to more favorable product evaluations [38]. High-quality content also promotes deeper cognitive processing and emotional engagement, enhancing consumer satisfaction and the likelihood of information adoption [39].

While an abundance of information can sometimes lead to overload, it often enhances perceived credibility by reflecting widespread acceptance and social validation of the product [40]. A large volume of eWOM provides diverse perspectives and reduces uncertainty, thereby aiding in more informed decision-making [38]. Moreover, information credibility, which includes the perceived trustworthiness and expertise of the source, plays a critical role in the persuasive impact of eWOM [41]. Credible eWOM increases consumer trust and positively influences attitudes and behaviors [42]. Expertise and the ability to verify the source’s authenticity further enhance credibility, facilitating deeper cognitive processing and fostering long-term changes in consumer attitudes and behaviors [43].

#### 2.2.2. The Organism (O).

The “Organism” (O) component in the SOR model involves the internal processing that transforms external stimuli into behavioral outcomes [19]. In the context of visual eWOM, this internal processing includes key constructs such as information usefulness, information adoption, and attitude toward information. Information usefulness assesses how consumers perceive eWOM as beneficial for enhancing their knowledge and aiding decision-making [32]. The perceived usefulness of visual eWOM is influenced by its quality, relevance, and comprehensiveness; high-quality eWOM significantly enhances decision-making effectiveness [44]. When consumers perceive eWOM as useful, it positively affects their cognitive evaluation and increases the likelihood of adopting and integrating this information into their decision-making processes [38].

Information adoption refers to the process by which consumers recognize, evaluate, and incorporate eWOM into their choices [45], which can significantly impact consumer behavior, leading to increased purchase intentions and a greater tendency to share eWOM [46]. Attitude toward information encompasses judgments about the relevance, trustworthiness, clarity, and emotional resonance of eWOM. A positive attitude toward eWOM enhances its perceived usefulness, leading to greater engagement and higher rates of adoption [47]. Consumers with a favorable attitude are more likely to integrate eWOM into their decision-making, leading to stronger purchase intentions and higher conversion rates [41]. A positive attitude also fosters trust and reduces skepticism, amplifying the credibility and impact of eWOM. Thus, understanding these factors of information usefulness, information adoption, and attitude toward information provides valuable insights into how visual eWOM influences consumer behavior within the SOR framework.

#### 2.2.3. The Response (R).

In the SOR model, the “response” (R) represents the final behavioral outcomes resulting from the organism’s internal processes. This response reflects an individual’s actions or reactions to the stimuli, shaped by their internal states [19]. In the context of eWOM, responses often manifest as consumer behaviors influenced by visual and informational stimuli. Purchase intention is a key response, where consumers form decisions to purchase a product or service based on the visual content they encounter. Vivid and interactive visual content has been shown to capture consumer attention and elicit positive emotions, thereby increasing their willingness to make a purchase [48]. High-quality images contribute to a sense of realism and reliability, fostering consumer trust and elevating the perceived credibility of the information [49].

Recent studies underscore the significance of visual cues in determining the usefulness of a message and influencing the behavioral intentions of readers [50]. Visual elements shape purchase intentions by enhancing the perceived value and reliability of eWOM content. Additionally, eWOM adoption can be driven by affective and curiosity perspectives, suggesting that beyond credibility, affective stimuli such as appealing visuals can propel consumers toward adopting eWOM and forming purchase intentions [39]. These affective reactions, influenced by visual eWOM, are crucial in shaping consumer behavior and motivating purchase decisions.

### 2.3 Hypothesis development

#### 2.3.1. Visual eWOM’s information quality impact on information usefulness, information adoption, attitude toward information.

The quality of information (INL) in visual electronic word of mouth (eWOM) is a critical determinant of its impact on consumer behavior. High-quality information, characterized by accuracy, relevance, comprehensiveness, and clarity, significantly enhances consumer trust, perceived usefulness, and purchase intentions [51]. Relevant visual content enables users to efficiently locate necessary information, thereby enhancing its utility for decision-making processes [38]. In a fast-paced digital environment, the recency of information significantly impacts its relevance and usefulness [52]. For instance, in industries such as fashion or technology, trends, and product features evolve rapidly, and having the latest visual content allows consumers to stay informed about the most recent developments. This timeliness ensures that the information users rely on is not outdated, thereby maintaining its applicability and enhancing its value in the decision-making process [53].

Clear visual content minimizes cognitive load, allowing users to quickly grasp the information without expending excessive mental effort [54]. This ease of understanding enhances the user experience and increases the likelihood of information being perceived as useful and credible. In the context of eWOM, this might involve detailed product images from multiple angles, step-by-step instructional videos, or infographics that summarize extensive data into digestible formats [55]. Comprehensive visual content ensures that consumers have access to all necessary information, reducing the risk of making uninformed or misinformed decisions. Xu [56] found that well-researched and objective information, especially when supported by high-quality visuals, boosts the perceived reliability of eWOM messages. When consumers trust the accuracy of information, they are more inclined to find it useful for making informed purchase decisions [57]. According to Nilashi et al. [58], high-quality information in visual eWOM should address user needs and interests. This focus on user needs ensures the information is directly applicable to consumers’ decision-making processes, thereby increasing its perceived usefulness [59]. Therefore, the following hypothesis is formulated:

**H1** Visual eWOM’s information quality has a positive impact on information usefulness.

In visual eWOM, high-quality visual information serves as a persuasive force, encouraging consumers to trust, utilize, and integrate the presented information into their decision-making processes [60]. The quality of visual eWOM significantly impacts cognitive and emotional evaluations, which in turn lead to positive behavioral responses, such as increased information adoption and higher purchase intentions [61]. High-quality visual eWOM enhances perceived credibility by presenting information in a polished and professional manner, fostering greater trust among users [62]. Visually appealing eWOM can engage users more effectively, evoking positive emotional responses that further increase the likelihood of information adoption [63]. The aesthetic appeal of well-designed visuals not only captures attention but also makes the information more memorable. This memorability is crucial, as it facilitates better recall of key details and benefits of the products or services discussed in the eWOM [64]. The ability to recall information easily due to its visual appeal and clarity enhances the chances of integrating it into the decision-making process. Moreover, consumers often perceive higher value in well-designed visual eWOM, which is influenced by the quality and relevance of the information as well as its overall presentation. This perception of value and trustworthiness encourages consumers to adopt the information and use it as a foundation for their decisions [65]. Consequently, the following hypothesis is formulated:

**H2** Visual eWOM’s information quality has a positive impact on information adoption.

The SOR theory suggests that high-quality visual eWOM, as a stimulus, enhances cognitive and emotional evaluations, leading to positive behavioral responses such as increased information adoption and higher purchase intentions [19]. High-quality visual eWOM also plays a crucial role in shaping users’ attitudes. The use of effective design elements, such as clear imagery, well-organized layouts, and legible typography, enhances the clarity and comprehensibility of the information [62]. When information is presented in a clear and easily digestible manner, it reduces cognitive load, making it easier for consumers to understand and process the content. This ease of comprehension leads to a more positive attitude toward the information, as users find it more useful and engaging [42]. Additionally, visually appealing eWOM can evoke positive emotional responses, such as enjoyment or satisfaction, further enhancing the likelihood of a favorable attitude. These positive emotions make the information more memorable and enjoyable, increasing the chances of consumers adopting the information and acting upon it [63]. Therefore, high-quality visual eWOM positively influences users’ attitudes by enhancing perceived credibility, fostering trust, and evoking positive emotional responses. This improved attitude increases the likelihood of information adoption and subsequent behavioral responses, such as purchasing decisions. Thus, the following hypothesis is established:

**H3** Visual eWOM’s information quality has a positive impact on attitude towards information.

#### 2.3.2. Visual eWOM’s information quantity impact on information usefulness, information adoption, attitude toward information.

In the context of visual eWOM, the concept of information quantity (INT) encompasses the volume of content, including comments, reviews, and video footage, shared by consumers about products or services on digital platforms [17]. A greater quantity of information can enhance its perceived usefulness by providing a more comprehensive and nuanced understanding of a topic [66]. For instance, eWOM, rich in visual content, such as multiple images and videos, is often perceived by consumers as more informative and helpful in decision-making processes [67]. When consumers are exposed to a large volume of information, they can access diverse perspectives and contexts, allowing for a thorough exploration of the subject, reducing uncertainty, and increasing confidence in the information’s accuracy [68].

The abundance of available information enhances users’ comprehension by enabling them to explore different dimensions and complexities of a topic. Exposure to diverse perspectives enables consumers to critically evaluate the relevance and applicability of information, ultimately helping them extract the most pertinent insights tailored to their specific needs [69]. John and De’Villiers [70] suggest that consumers are more likely to actively engage with visual content, leading to a deeper understanding of the product and a greater propensity to adopt the information conveyed in the visual eWOM message. Interaction with visual elements not only improves comprehension but also fosters a sense of trust and reliability, especially when consumers witness products being used or see others’ positive experiences [37]. Based on this reasoning, the following hypothesis is proposed:

**H4** Visual eWOM’s information quantity has a positive impact on information usefulness.

The quantity of information available can significantly influence consumers’ propensity to adopt it. When users are exposed to a large volume of detailed and relevant information, they can gain a comprehensive understanding of the subject matter, which is crucial for informed decision-making [71]. This extensive information enhances the source’s credibility, as users tend to trust content-rich sources. Moreover, the availability of abundant information helps reduce uncertainty and ambiguity, allowing users to feel more confident in their decisions [72]. Visual elements, such as images and videos, play a vital role in clarifying complex product features and functionalities, making the information more accessible and comprehensible [73]. Users often perceive more substantial value in extensive information, believing that it offers deeper insights and knowledge [74].

This comprehensive information promotes greater engagement and involvement, which leads to deeper exploration and understanding of the content [75]. Such engagement aids decision-making by providing the necessary justification for users’ choices, thereby increasing the likelihood of information adoption [76]. Ultimately, ample information enhances cognitive processing, allowing users to more effectively process and internalize the content. As a result, users are more likely to adopt the information when they feel well-informed, confident, and supported by the depth and breadth of the available content [77]. Hence, the following hypothesis is proposed:

**H5** Visual eWOM’s information quantity has a positive impact on information adoption.

When sufficient information is available, it satisfies the curiosity of individuals seeking knowledge, resulting in a more favorable attitude toward the information source [78]. Access to abundant information enables individuals to make more informed choices, which boosts their confidence and fosters a positive attitude [79]. This abundance can also alleviate cognitive dissonance by helping individuals reconcile conflicting viewpoints, resulting in a more favorable perception [80]. Moreover, the availability of a large quantity of information promotes prolonged engagement, which deepens the connection with the material and enhances the attitude towards it [81].

According to Karami [82], a higher quantity of well-chosen visuals, such as images and videos, can foster more positive attitudes by facilitating deeper mental processing of the content. The extensive availability of information enables individuals to support their arguments or viewpoints, thereby strengthening their positive attitudes [83]. When consumers encounter a greater volume of visual eWOM, they are more likely to compare different sources, cross-check information, and validate their perspectives. The inclusion of diverse perspectives broadens understanding and appreciation, contributing to a more positive overall attitude [46]. Mathur et al. [59] suggest that images and videos showcasing a product’s appeal or positive user experiences can evoke positive emotions, ultimately fostering a more favorable attitude towards the eWOM content and potentially influencing purchase decisions. Thus, the quantity of information not only satisfies curiosity and reduces cognitive dissonance but also fosters deeper engagement, supports argumentation, and incorporates diverse perspectives, all contributing to a more positive attitude towards the information [84]. Consequently, the following hypothesis is formulated:

**H6** Visual eWOM’s information quantity has a positive impact on attitude towards information.

#### 2.3.3. Visual eWOM’s information credibility impact on information usefulness, information adoption, attitude toward information.

Information credibility (INC), is defined by Tseng and Fogg [85] as the perceived believability of information, influenced by trustworthiness and expertise. These elements are crucial in determining how users evaluate and respond to visual eWOM information. Trustworthy and expert visual content significantly enhances cognitive responses and increases the perceived usefulness of the information. When visual eWOM originates from reliable sources, it boosts the perceived reliability of the information, making users more inclined to trust and act on it [86]. Authentic visuals, such as user-generated content that showcases real-life experiences, reduce skepticism and elevate the perceived value of the information [87]. High-quality visuals that clearly detail products and user experiences further contribute to the clarity and utility of the information [88]. Additionally, credible visuals often have higher persuasive power due to their aesthetic appeal, effectively showcasing a product’s value or service’s value. These visuals engage users more effectively, capturing their attention and fostering greater interaction, which enhances information retention and perceived usefulness [89]. Furthermore, visual eWOM from reputable sources provides social proof, validating positive experiences and reinforcing the information’s utility. By ensuring the credibility of visual eWOM, content creators and marketers can significantly enhance the perceived usefulness of their information, resulting in better-informed consumers and more effective communication [37]. Based on the literature, the following hypothesis is developed:

**H7** Visual eWOM’s information credibility has a positive impact on information usefulness.

Credible sources with established reputations for honesty amplify the trustworthiness of the information, leading to a higher likelihood of adoption [36]. Visual content provides tangible and authentic representations of products or services, which effectively mitigates uncertainty and builds user confidence in the information presented [90]. For instance, a video demonstrating a product’s use can provide a clearer, more convincing portrayal than text alone. Additionally, visual eWOM can evoke emotional responses that strengthen the perceived credibility of the information. Engaging and high-quality visuals are capable of eliciting positive emotions, which in turn can influence users’ attitudes toward the content [91]. Social proof, indicated by metrics such as likes, shares, or comments, reinforces the credibility of the information [43]. High engagement levels signal to potential adopters that the information is valued by others, thereby increasing its perceived relevance [92]. Moreover, visuals often simplify complex information, making it more accessible and comprehensible, which in turn facilitates easier adoption [93]. When visual eWOM aligns with users’ prior expectations or experiences, it reinforces the credibility of the content, as consistency with previously encountered information validates its accuracy and reliability [94]. Thus, the following hypothesis is proposed:

**H8** Visual eWOM’s information credibility has a positive impact on information adoption.

Credible visual eWOM, characterized by high-quality images, videos, or infographics from authoritative or reliable sources, fosters a sense of trustworthiness among users [91]. This trust is fundamental as it supports the perceived accuracy and reliability of the information, making users more receptive and positive towards it [56]. Additionally, engaging and professionally produced visual content captures users’ attention more effectively than textual information, thereby improving their understanding and retention [95]. The principle of social proof further reinforces this effect, as endorsements or shared experiences from trusted or influential figures lend additional credibility to the visual content [96]. Furthermore, the emotional resonance of well-crafted visuals can elicit positive emotional responses, further enhancing users’ attitudes [91]. Overall, the integration of credibility into visual eWOM facilitates a more favorable attitude toward the information by combining trust, accuracy, engagement, social validation, and emotional impact [97]. Therefore, the following hypothesis is proposed:

**H9** Visual eWOM’s information credibility has a positive impact on attitude towards information.

#### 2.3.4. Visual eWOM’s information usefulness impact on information adoption and online purchase intention.

Information usefulness (INU) plays a crucial role in shaping consumer engagement with information systems, as it helps individuals process and evaluate relevant content, ultimately aiding them in making well-informed decisions [98]. When information is perceived as useful, consumers are more likely to invest time and effort in understanding and utilizing it, reinforcing its impact on their decision-making processes [98]. In the context of visual eWOM, the perceived usefulness of visual content such as user-generated images, videos, and reviews plays a significant role in shaping consumer behavior. When users find visual eWOM to be useful, it enhances their trust in the information presented, which, in turn, increases the likelihood of adopting the information [86]. Useful visual eWOM that effectively reduces uncertainty about a product or service by showcasing real-life user experiences, through elements like social proof, contributes to this trust and adoption [36]. This validation from other users can positively influence information adoption. Furthermore, the superior processing and retention of visual information compared to text-based content bolsters its effectiveness. The human brain’s ability to process and remember visual information more efficiently means that useful visual eWOM aids in the recall of key product details, which supports information adoption [99]. Empirical evidence highlights the substantial impact of visual eWOM on adoption rates, emphasizing its role in simplifying complex information and enhancing perceived authenticity through visual elements [100]. Based on these insights, the following hypothesis is proposed:

**H10** Visual eWOM’s information usefulness has a positive impact on information adoption.

The usefulness of visual eWOM significantly influences online purchase intentions by addressing perceived risk and enhancing trust and credibility. According to González-Rodríguez et al. [86], the usefulness of eWOM information plays an important role in shaping online purchase intention. Detailed and informative visual eWOM, including reviews and testimonials, clarifies product quality and performance, thereby making consumers feel more secure about their purchasing decisions [71]. Additionally, when visual eWOM effectively showcases the benefits and advantages of a product, it enhances the perceived value of the product, leading to a greater likelihood of purchase [101]. Consumers are more likely to return to online stores if they find the eWOM information helpful in making informed decisions [102]. Similarly, Xiao et al. [67] found that consumers are more inclined to consider a product worth purchasing when they clearly understand its value proposition through useful eWOM. Furthermore, when consumers perceive the information as credible and trustworthy, they tend to trust the brand and develop a positive attitude toward purchasing [103]. By mitigating perceived risks and building credibility, useful visual eWOM significantly boosts online purchase intentions, contributing to a positive consumer experience and encouraging repeat visits to online platforms [46]. Therefore, the following hypothesis is established:

**H11** Visual eWOM’s information Usefulness has a positive impact on Online Purchase Intention.

#### 2.3.5. Attitude towards visual eWOM’s information impact on information adoption.

Attitude toward information (ATI) is a critical determinant in shaping individuals’ responses to messages, including eWOM. Attitude is defined by Eagly and Chaiken [104] as a psychological tendency expressed by evaluating a particular entity with some degree of favor or disfavor. When eWOM content aligns with an individual’s specific needs and interests, it fosters a more positive attitude, subsequently enhancing engagement and increasing the likelihood of information adoption [105]. Furthermore, when information is presented in a well-structured, detailed, and easily understandable manner, it facilitates cognitive processing by reducing ambiguity and enhancing comprehension [106]. A well-organized presentation allows individuals to systematically analyze the credibility and relevance of the content, increasing their confidence in the information provided.

Moreover, emotional and social influences further impact attitudes and adoption. eWOM that elicits emotional responses, or comes from trusted peers and social circles, can significantly strengthen positive attitudes [107]. Positive emotional connections and social proof, such as endorsements from friends or influencers, enhance the likelihood of information adoption [108]. The consistency and frequency of eWOM exposure also reinforce positive attitudes. Regular exposure to consistent, positive messaging solidifies user attitudes, fostering habitual reliance on eWOM for decision-making [102]. Platforms like Instagram and YouTube are particularly effective due to their ability to capture attention and evoke strong emotional responses through visual content. These platforms facilitate storytelling through images and videos, making the information more relatable, compelling, and easier to adopt [109]. Based on these insights, the following hypothesis is proposed:

**H12** Attitude toward visual eWOM’s information has a positive impact on information adoption.

Favorable attitudes towards eWOM can evoke positive emotional responses, such as enthusiasm or excitement, which increase the consumer’s motivation to buy [45]. A positive attitude towards eWOM not only enhances the perceived value of the information but also elevates the perceived value of the product or service, making consumers more likely to act on the information [63]. Additionally, by mitigating perceived risks associated with online purchasing such as concerns about product quality or service reliability a positive attitude towards eWOM reassures consumers, alleviating their apprehensions and increasing their likelihood of making a purchase [37].

The positive attitude towards eWOM effectively bridges the gap between trust, perceived value, emotional engagement, social proof, and risk reduction, leading to a heightened intention to purchase online. Studies employing the SOR framework have demonstrated that visual stimuli in eWOM can evoke positive emotional responses, enhancing the persuasiveness of the information and increasing purchase likelihood [32]. Furthermore, repetitive exposure to positive eWOM fosters familiarity and comfort with the product or service, which reinforces purchase intention through increased familiarity [110]. Consumers who develop a positive perception of eWOM are more likely to engage in further research or exploration of the product, thereby increasing their readiness and intention to make a purchase [111]. Therefore, the following hypothesis is proposed:

**H13** Attitude toward visual eWOM*’s* information has a positive impact on online purchase intention.

#### 2.3.6. Visual eWOM’s information adoption impact on online purchase intention.

Information adoption (INA) refers to the process through which individuals actively evaluate, internalize, and apply information to guide their decision-making. This process requires assessing the credibility, relevance, and usefulness of the information before incorporating it into their cognitive framework [112]. In the context of visual eWOM, this process is crucial for influencing consumer behavior, particularly online purchase intention. When consumers adopt information presented in visual eWOM in their knowledge base, they often gain enhanced knowledge and active recall about a product’s features and benefits. This increased understanding empowers them to make informed decisions, bolstering their confidence and subsequently increasing their likelihood of making a purchase [38].

The emotional connection fostered by visual eWOM is another critical factor. Personal stories and experiences shared through eWOM can evoke strong emotional responses, thereby strengthening consumers’ purchase intentions [113]. Additionally, the comparative advantage highlighted in positive eWOM where the product’s superiority over competitors is emphasized can sway consumers towards choosing the featured product [114]. The perceived popularity of a product, as indicated by a large volume of positive eWOM, can create a bandwagon effect, compelling consumers to purchase to align with the majority [115].

Detailed customer testimonials provide practical insights into real-life product performance, which are often more persuasive than generic product descriptions. These testimonials help build trust and, consequently, enhance purchase intention [116]. Moreover, interactive engagement on platforms with Q&A sections allows potential buyers to ask questions and receive real-time feedback. This immediate clarification of doubts reinforces purchase decisions, making consumers more comfortable with committing to a purchase [117]. Therefore, the adoption of information from visual eWOM not only enriches consumers’ knowledge and emotional connection but also leverages social proof and interactive engagement to solidify purchase intentions. Hence, the following hypothesis is proposed:

**H14** Visual eWOM’s information adoption has a positive impact on online purchase intention.

### 2.4. Research framework

**Fig 1:**
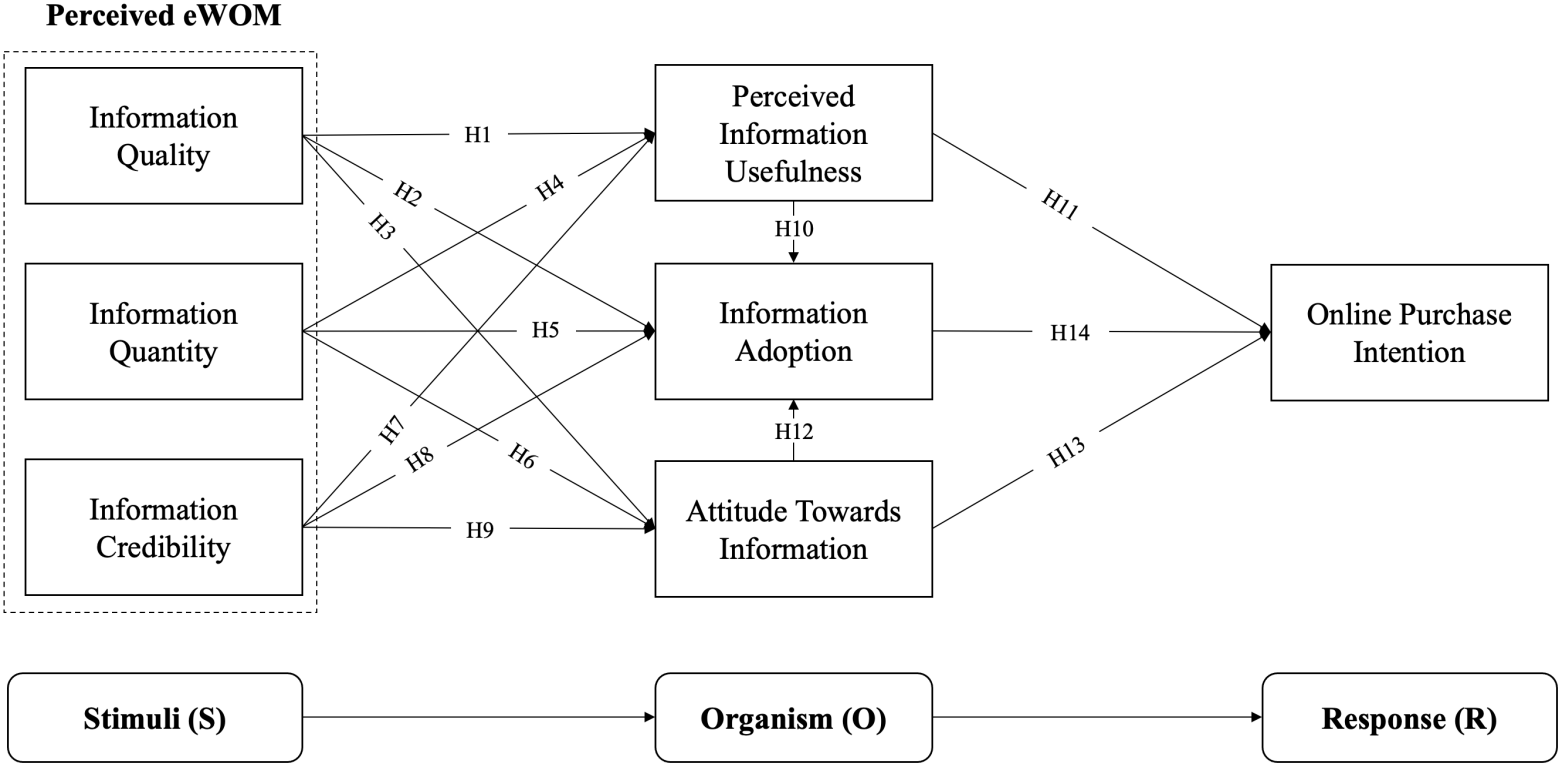
Research framework.

Drawing upon the reviewed literature, the proposed hypotheses and the theoretical framework of this study are presented in Fig 1. This framework integrates key constructs such as information quality, quantity, and credibility of visual eWOM, as well as their impact on information usefulness, information adoption, attitude towards information, and online purchase intention. By linking these constructs, the framework illustrates how different aspects of visual eWOM contribute to consumers’ decision-making processes. It highlights the pathways through which visual eWOM influences consumer behavior, providing a comprehensive understanding of the mechanisms at play. This theoretical model serves as a foundation for empirically testing the relationships among these variables, thereby advancing our knowledge of the effectiveness of visual eWOM in shaping online consumer behavior.

## 3. Methodology

### 3.1. Measurement

The measurements utilized in this study were adapted from established scales in the literature to ensure content validity. The original questionnaire was developed in English and subsequently translated into Vietnamese using the back-translation method recommended by Brislin [118]. The translation process followed a rigorous approach to ensure linguistic and conceptual accuracy across both the pilot study and the final survey. First, a bilingual expert translated the survey from English to Vietnamese. To verify the accuracy of this translation, an independent bilingual expert—who had no prior exposure to the original English version—conducted a back-translation into English. The two English versions were then carefully compared to identify any discrepancies, with necessary adjustments made to preserve the intended meaning of each item. To further enhance clarity and comprehensibility, the translated questionnaire underwent a pretest through a pilot study. Participants provided feedback on the wording, cultural appropriateness, and overall readability of the survey items. Based on their insights, minor refinements were implemented to ensure that all questions were easily understood and effectively captured the intended constructs. This standardized translation procedure was applied consistently across both the pilot study and the final survey, ensuring reliability and validity while maintaining cultural relevance.

The constructs measured in this study included information quality (INL), information quantity (INT), information credibility (INC), information usefulness (INU), information adoption (INA), attitude towards information (ATI), and online purchase intention (OPI). Information quality was assessed using a four-item scale adapted from [119–121]. Information quantity was evaluated with a two-item scale adapted from [122]. Information credibility was measured using a four-item scale derived from [120,123,124]. Information usefulness was measured using a three-item scale adapted from [119,123,125,126]. Information adoption was assessed with a three-item scale adapted from [127]. Attitude towards information was measured using a three-item scale adapted from [121], and online purchase intention was assessed using a three-item scale adapted from [128]. The complete structure of the questionnaire, including the items for each construct, is detailed in Table 2.

**Table 1:** Respondents’ information and social media usage.

Respondents’ Information (N = 335)		Frequency	Percentage
Gender	Male	183	54.6
	Female	146	43.6
	Other	6	1.8
Age	18-21	118	35.2
	22-25	199	59.4
	Over 25	18	5.4
The most used platform	Facebook	185	55.2
	TikTok	93	27.8
	YouTube	42	12.5
	Instagram	15	4.5
Used time per day	Less than 1 hour	60	17.9
	From 1 hour to 2 hours	100	29.8
	From 2 hour to 3 hours	81	24.2
	More than 3 hours	94	28.1

**Table 2:** Convergent validity.

Constructs’ Indicators	Loading	Cronbach’s Alpha	Composite Reliability (CR)	Average Variance Extracted (AVE)
Information Quality (INL)		0.792	0.863	0.613
INL1: “I can understand visual eWOM information shared in social media.”	0.820			
INL2: “I think visual eWOM information shared in social media is clear.”	0.682			
INL3: “I find visual eWOM information in social media relevant to my needs.”	0.833			
INL4: “I think visual eWOM information in social media is detailed.”	0.787			
Information Quantity (INT)		0.823	0.918	0.849
INT1: “I can rely on the amount of visual eWOM information in social media.”	0.833			
INT2: “The amount of visual eWOM information in social media can help me understand the product performance.”	0.806			
Information Credibility (INC)		0.828	0.885	0.658
INC1: “I think visual eWOM information in social media is convincing.”	0.881			
INC2: “I think visual eWOM information in social media is credible.”	0.880			
INC3: “I think visual eWOM information in social media is believable.”	0.861			
INC4: “I think visual eWOM information in social media is trustworthy.”	0.894			
Information Usefulness (INU)		0.740	0.852	0.657
INU1: “Visual eWOM information in social media is generally useful for me to evaluate the product.”	0.823			
INU2: “Visual eWOM information in social media is generally helpful for me to evaluate the product.”	0.773			
INU3: “Visual eWOM information in social media is generally informative for me to evaluate the product.”	0.836			
Information Adoption (INA)		0.769	0.866	0.684
INA1: “I learn something new about brands through visual eWOM information in social media.”	0.796			
INA2: “I accept the visual eWOM information of brands in social media.”	0.843			
INA3: “I accept the visual eWOM recommendation of brands in social media.”	0.841			
Attitude Towards Information (ATI)		0.777	0.870	0.691
ATI1: “I always review visual eWOM information in social media when I want to buy a product.”	0.833			
ATI2: “I find visual eWOM information in social media helpful for me to make decisions when buying a product.”	0.826			
ATI3: “I feel visual eWOM information in social media makes me more confident in purchasing a product.”	0.835			
Online Purchase Intention (OPI)		0.752	0.856	0.664
OPI1: “It is very likely that I will buy the product.”	0.807			
OPI2: “I will buy the product next time I need a product.”	0.772			
OPI3: “I will recommend the product to my friends.”	0.864			

### 3.2. Data collection

This study employed a quantitative research approach to explore the impact of perceived visual eWOM on purchase intention. A non-probability convenience sampling method was used, as it offered cost-effectiveness and allowed the researchers to efficiently reach a large number of relevant respondents within the given timeline [129]. The sampling strategy specifically targeted active social media users who frequently engaged with online visual content, such as liking, sharing, commenting, and making purchase decisions influenced by visual eWOM. To ensure participant relevance, eligibility criteria required respondents to be at least 18 years old, actively follow or engage with brands on social media platforms, and have prior experience evaluating products based on user-generated visual content. The survey questionnaire was organized into three main sections. The first section collected demographic information, including age and gender. The second section focused on participants’ social media usage, identifying their most frequently used platforms and the frequency of their online shopping activities. Finally, the third section measured the study’s seven key constructs using a 5-point Likert scale (1 = strongly disagree, 5 = strongly agree).

The data collection was carried out in March 2024 through an online questionnaire distributed via Google Forms. The survey link was shared across various platforms, including Facebook, Zalo, and Instagram, to engage individuals involved in online shopping and interactions with visual eWOM. To ensure the instrument’s validity and reliability, a pilot study involving 50 participants was conducted from March 1st to 5th, 2024. Participants were provided with a clear overview of the study’s objectives and purpose before completing the survey. To ensure a consistent understanding of visual eWOM, they received essential background information on key elements, including influencer endorsements and brand-created promotional content in the form of images and short videos. These materials were drawn from widely used social media platforms such as Facebook, Instagram, TikTok, and YouTube. This approach ensured that all participants had adequate exposure to and comprehension of visual eWOM, allowing them to engage with the survey content in an informed and meaningful way. To refine the participant pool and enhance data reliability, an initial set of screening questions was included in both the pilot study and the main survey. One such screening question was: *“Do you participate in visual eWOM activities on social networking sites?”* This step ensured that only individuals with relevant experience were included in the study, thereby improving the validity of the findings. The internal consistency of the scales was measured using Cronbach’s alpha (CA), with values ranging from 0.702 to 0.812, exceeding the recommended threshold of 0.7 [130], thereby confirming the instrument’s reliability for the main study. Following the pilot phase, the main data collection took place from March 9th to 30^th^, 2024. A total of 417 responses were initially collected. After screening for incomplete or illogical responses and excluding those who never engaged in social media or online shopping, 335 valid responses remained. This sample size exceeds the minimum requirement for structural equation modeling (SEM), which recommends at least 10 times the number of items in the model [131]. Therefore, the final sample ensured adequate statistical power for the analysis.

Participation in the study was voluntary, and informed consent was obtained before respondents could proceed with the survey. Consent was provided by selecting a checkbox in the online questionnaire, confirming that participants agreed to take part willingly and understood that their anonymized data would be used exclusively for research purposes. The objectives of the study were clearly outlined at the start of the survey, emphasizing the confidentiality and anonymity of all responses. The study received ethical approval (Approval No. 20240108.02) from the Board of Directors at FPT Can Tho University, Vietnam. All research procedures complied with the university’s ethical standards and guidelines for studies involving human participants.

### 3.3. Participants

The study’s sample consisted of 335 participants engaged in visual eWOM activities on social media platforms, including following, liking, sharing, commenting, and interacting with visual content. The sample was predominantly male, with 183 participants (54.6%), while females constituted 146 participants (43.6%), and 6 participants (1.8%) identified as ‘other’. The majority of respondents (59.4%) were aged between 22 and 25 years, while 35.2% (118 participants) were aged between 18 and 21 years. In terms of social media usage, Facebook emerged as the most commonly used platform, with 185 participants (55.2%) identifying it as their primary platform. TikTok, YouTube, and Instagram followed, with 27.8%, 12.5%, and 4.5% of participants, respectively. The time spent on social media varied among participants, with the largest group (29.8%) spending 1–2 hours per day, followed by 28.1% spending more than 3 hours, 24.2% spending 2–3 hours, and 17.9% spending less than 1 hour per day. The demographic information of participants is presented in Table 1.

### 3.4. Data analysis

The research employed Partial Least Squares Structural Equation Modeling (PLS-SEM) for data analysis, using SmartPLS software. PLS-SEM was chosen for its suitability in exploratory research, ability to handle complex models, and robustness with non-normal data distributions [132]. The analysis followed a two-step approach as recommended by Hair et al. [133]. First, the measurement model was assessed for reliability and validity, including individual item reliability, construct reliability, convergent validity, and discriminant validity. Second, the structural model was evaluated by examining the path coefficients, variance inflation factor (VIF), coefficient of determination (R²), and predictive relevance (Q²). These analyses provided a comprehensive evaluation of the research model and hypotheses, offering insights into the relationships between perceived visual eWOM characteristics and purchase intention.

## 4. Results

The study’s results are presented in two main sections: measurement model analysis and structural model analysis. The measurement model analysis was conducted to verify the validity and reliability of the constructs, while the structural model analysis tested the hypothesized relationships between perceived visual eWOM characteristics and purchase intention, as well as evaluated the overall effectiveness of the proposed research model.

### 4.1. Measurement model analysis

#### 4.1.1. Internal consistency reliability.

To evaluate the reliability and accuracy of the measurement instruments, several metrics were employed, including Cronbach’s alpha, composite reliability (CR), and average variance extracted (AVE). The results, presented in Table 2, indicate that all constructs exhibited satisfactory levels of internal consistency and convergent validity. Cronbach’s alpha values for the constructs ranged from 0.740 to 0.828, exceeding the threshold of 0.7, which suggests that the constructs have a satisfactory level of internal consistency [130]. Additionally, all item loadings were high, ranging from 0.772 to 0.937, surpassing the threshold of 0.5 [134]. This indicates a strong relationship between the items and their respective latent variables. However, it was noted that the loading for INL2 was slightly lower (0.682) compared to other indicators, suggesting a relatively weaker association with the underlying latent variable. Moreover, all constructs demonstrated AVE values above the recommended threshold of 0.5, and the CR values exceeded 0.7, further confirming the strong convergent validity of the measurement model [134,135]. These findings collectively suggest that the indicators used in the study adequately represent their respective constructs.

#### 4.1.2. Discriminant validity.

Construct validity was further assessed using the Fornell-Larcker criterion and Heterotrait-Monotrait (HTMT) ratios, with results detailed in Tables 3 and 4, respectively. Table 3 presents the Fornell-Larcker criterion results, which assess discriminant validity by comparing the square root of the average variance extracted (AVE) with the correlations between different constructs. The analysis confirmed that for each construct, the square root of its AVE exceeded its correlations with other constructs, with diagonal values ranging from 0.783 to 0.921, all of which exceeded the highest inter-construct correlation of 0.419. This result demonstrates that the constructs are distinct from each other [136]. Additionally, Table 4 shows that the HTMT ratios were all below the recommended threshold of 0.90, with values ranging from 0.139 to 0.521, further confirming the good discriminant validity between the constructs [137]. These findings collectively provide strong evidence for the discriminant validity of all constructs in the study, reinforcing the overall construct validity of the measurement model [133].

**Table 3:** Fornell-larcker criterion.

	ATI	INA	INC	INL	INT	INU	OPI
**ATI**	**0.831**						
**INA**	0.333	**0.827**					
**INC**	0.419	0.390	**0.811**				
**INL**	0.185	0.289	0.331	**0.783**			
**INT**	0.269	0.289	0.372	0.317	**0.921**		
**INU**	0.209	0.320	0.310	0.258	0.322	**0.811**	
**OPI**	0.261	0.408	0.275	0.212	0.110	0.168	**0.815**

*Note:* INL = Information Quality, INT = Information Quantity; INC = Information Credibility; INU = Information Usefulness; INA = Information Adoption; ATI = Attitude Towards Information; OPI = Online Purchase Intention.

**Table 4:** HTMT (Heterotrait-Monotrait ratio).

	ATI	INA	INC	INL	INT	INU	OPI
**ATI**							
**INA**	0.429						
**INC**	0.496	0.478					
**INL**	0.216	0.362	0.394				
**INT**	0.335	0.357	0.441	0.368			
**INU**	0.278	0.422	0.393	0.330	0.406		
**OPI**	0.339	0.521	0.337	0.257	0.139	0.217	

*Note:* INL = Information Quality, INT = Information Quantity; INC = Information Credibility; INU = Information Usefulness; INA = Information Adoption; ATI = Attitude Towards Information; OPI = Online Purchase Intention.

### 4.2 Structural model assessment

#### 4.2.1. Assessment of comment method bias.

The presence of strong correlations among observed variables can indicate common method bias, potentially compromising the validity of the scale. In causal models, high levels of multicollinearity among observed variables can distort analytical outcomes and lead to questionable interpretations. To address these concerns, this study employed the Variance Inflation Factor (VIF) as a diagnostic tool, in line with the recommendations of Kock [138]. The VIF values for all relationships in the model were carefully examined, with results presented in Table 5 showing values ranging from 1.129 to 1.430. These values fall well below the commonly accepted threshold of 5 [138], suggesting that multicollinearity is not a significant issue in this model. The highest VIF (1.430) is observed in the relationship between INC and INA, while the lowest (1.129) is associated with the relationship between INU and OPI. These findings support the discriminant validity of the constructs and reinforce the overall reliability of the structural model’s results.

**Table 5:** Results of testing hypotheses in the theoretical framework.

Hypothesis	Structural	β	SD	T-statistics	P-values	VIF	Result
**H1**	INL - > INU	0.128	0.055	2.293	0.022	1.181	Accepted
**H2**	INL - > INA	0.125	0.051	2.429	0.015	1.201	Accepted
**H3**	INL - > ATI	0.025	0.052	0.482	0.630	1.181	Rejected
**H4**	INT - > INU	0.211	0.054	3.829	0.000	1.220	Accepted
**H5**	INT - > INA	0.076	0.057	1.358	0.175	1.289	Rejected
**H6**	INT - > ATI	0.126	0.058	2.198	0.028	1.220	Accepted
**H7**	INC - > INU	0.189	0.054	3.504	0.000	1.233	Accepted
**H8**	INC - > INA	0.197	0.062	3.068	0.002	1.430	Accepted
**H9**	INC - > ATI	0.364	0.052	7.025	0.000	1.233	Accepted
**H10**	INU - > INA	0.167	0.052	3.141	0.001	1.194	Accepted
**H11**	INU - > OPI	0.025	0.054	0.469	0.639	1.129	Rejected
**H12**	ATI - > INA	0.172	0.060	2.862	0.004	1.240	Accepted
**H13**	ATI - > OPI	0.138	0.063	2.237	0.025	1.140	Accepted
**H14**	INA - > OPI	0.354	0.054	6.515	0.000	1.215	Accepted

*Note:* INL = Information Quality, INT = Information Quantity; INC = Information Credibility; INU = Information Usefulness; INA = Information Adoption; ATI = Attitude Towards Information; OPI = Online Purchase Intention.

#### 4.2.2. Results of testing hypotheses.

**Fig 2:**
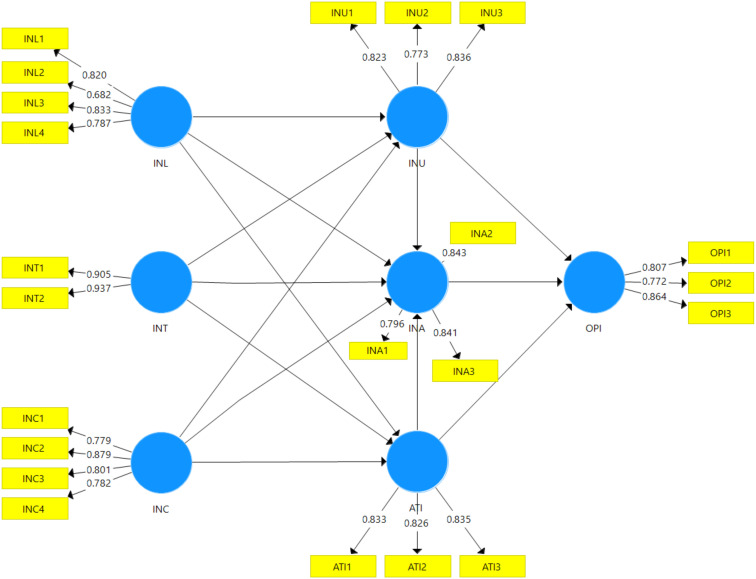
PLS-SEM Results (Structural Model).

The results of the hypothesis testing, presented in Table 5 and Fig 2, provide substantial support for most of the hypotheses, with the exceptions of H3, H5, and H11, which were not supported by the data. Specifically, hypotheses H1 and H2 were supported, indicating significant positive relationships between information quality (INL) and both information usefulness (INU) (β = 0.128, p = 0.022) and information adoption (INA) (β = 0.125, p = 0.015). These results suggest that high-quality visual information is processed more deeply, leading to lasting attitude changes. The positive impact on information adoption aligns with the concept of cognitive ease and fluency, where high-quality visuals defined by clarity, aesthetics, relevance, and coherence are inherently more engaging and easier to process. Consequently, such visuals evoke favorable cognitive and affective responses, making consumers more likely to perceive the information as useful and to adopt it. Conversely, hypothesis H3 was not supported, as information quality (INL) did not have a significant effect on attitude toward information (ATI) (β = 0.025, p = 0.630). This suggests that while high-quality visuals improve understanding and engagement, they may not directly influence overall attitudes toward the information. This also indicates that other factors such as credibility and source expertise might play a more dominant role in attitude formation.

Regarding information quantity (INT), the analysis results supported H4 and H6, showing significant positive relationships between information quantity (INT) and both information usefulness (INU) (β = 0.211, p = 0.000) and attitude (ATI) (β = 0.126, p = 0.028). These findings underscore the power of visual abundance in capturing consumer attention and shaping favorable perceptions, aligning with the mere exposure effect. Moreover, the positive relationship between information quantity (INT) and information usefulness (INU) suggests that a higher quantity of visuals can signal the information’s comprehensiveness and value, particularly in online environments where consumers have limited time to evaluate numerous options. However, H5 was not supported, as the quantity of information (INT) did not significantly affect information adoption (INA) (β = 0.076, p = 0.175). This finding underscores the limitations of depending solely on the quantity of visual content to drive behavioral change. It supports the concept of information overload, where an excessive amount of visual stimuli can overwhelm consumers and impede their ability to process information effectively.

Information credibility (INC) emerged as a particularly strong predictor, with all related hypotheses (H7, H8, and H9) being supported. Specifically, information credibility (INC) showed significant positive relationships with information usefulness (INU) (β = 0.189, p = 0.000), information adoption (INA) (β = 0.197, p = 0.002), and attitude (ATI) (β = 0.364, p = 0.000). These findings underscore the critical role of credibility in shaping consumer perceptions and behaviors. The strong relationship between information credibility (INC) and information adoption (INA) suggests that credible information is more likely to be internalized and integrated into consumers’ decision-making processes. Moreover, the influence of information credibility (INC) on attitude (ATI) is consistent with the SOR theory, indicating that credible stimuli evoke positive emotional responses, such as trust and confidence. These results emphasize the necessity of enhancing trust and credibility in visual eWOM strategies to effectively influence consumer attitudes and actions.

The analysis also supported H10, demonstrating a significant positive relationship between information usefulness (INU) and information adoption (INA) (β = 0.167, p = 0.001). This finding underscores the importance of perceived usefulness in driving information adoption. However, H11 was not supported, as information usefulness (INU) did not directly influence online purchase intention (OPI) (β = 0.025, p = 0.639). This suggests that usefulness alone may not be sufficient to drive purchase intentions, emphasizing the role of other factors moderating in shaping consumer choices.

Attitude towards information (ATI) demonstrated significant positive relationships with both information adoption (INA) (β = 0.172, p = 0.004) and online purchase intention (OPI) (β = 0.138, p = 0.025), supporting H12 and H13. These results underscore the critical role of attitudes in shaping information processing and decision-making. Specifically, the positive relationship between attitude (ATI) and online purchase intention (OPI) illustrates how emotional responses influence consumer behavior, demonstrating the impact of attitudes on driving purchase intentions. Finally, H14 was supported, showing a strong positive relationship between information adoption (INA) and online purchase intention (OPI) (β = 0.354, p = 0.000). This result indicates that information adoption is a crucial mediator between perceived visual eWOM characteristics and purchase intention.

#### 4.2.3. Evaluation of explanation and prediction power of research model.

In assessing the performance metrics of the research model, both explanatory power (R²) and predictive relevance (Q²) were evaluated for the endogenous constructs [139]. Table 6 outlines the R² and Q² values for online purchase intention (OPI), information usefulness (INU), information adoption (INA), and attitude towards information (ATI). The R² values ranged from 0.152 to 0.234, reflecting weak to moderate explanatory power based on Hair et al. [140] criteria. Information adoption (INA) demonstrated the highest R² at 0.234, indicating that the model explains 23.4% of its variance, while INU had the lowest R² at 0.152. The Q² values, which ranged from 0.102 to 0.159, all indicated modest predictive relevance for the constructs [141]. Information adoption (INA) again showed the strongest performance with a Q² of 0.159, whereas information usefulness (INU) had the lowest Q² at 0.102. These findings suggest that while the model offers useful explanatory and predictive insights, particularly for information adoption (INA), there may be other factors not captured by the current model that could further clarify the variance in the endogenous constructs.

**Table 6:** Performance metrics (R-squared, Q-squared).

Constructs	R^2^	Q^2^
OPI	0.177	0.109
INU	0.152	0.102
INA	0.234	0.159
ATI	0.184	0.126

*Note:* INU = Information Usefulness; INA = Information Adoption; ATI = Attitude Towards Information; OPI = Online Purchase Intention.

#### 4.2.4. Importance performance matrix analysis (IPMA).

The Importance Performance Matrix Analysis (IPMA) offers valuable insights into the relative importance and performance of latent variables in predicting online purchase intention (OPI) [142]. As presented in Table 7, information adoption (INA) emerges as the most critical factor, with the highest importance score (total effect) of 0.358 and a relatively high-performance level of 63.064. This suggests that information adoption (INA) is a significant driver of online purchase intention (OPI) and is relatively well-managed in the current context. Attitude towards information (ATI) is the second most important factor, with an importance score of 0.187 and a high-performance rating of 62.353.

**Table 7:** Importance performance matrix analysis (IPMA).

	Importance (Total effect of the latent variable online purchase intention)	LV Performances
**ATI**	0.187	62.353
**INA**	0.358	63.064
**INC**	0.165	44.370
**INL**	0.065	46.411
**INT**	0.056	57.774
**INU**	0.093	69.760

*Note:* INL = Information Quality, INT = Information Quantity; INC = Information Credibility; INU = Information Usefulness; INA = Information Adoption; ATI = Attitude Towards Information.

Interestingly, while information usefulness (INU) exhibits the highest performance score (69.760), its importance score (0.093) is relatively low. This discrepancy indicates that current practices might be overemphasizing the usefulness of information. Conversely, visual eWOM information credibility (INC) shows moderate importance (0.165) but the lowest performance score (44.370), highlighting a critical area for improvement. Visual eWOM information quality (INL) and information quantity (INT) have the lowest importance scores (0.065 and 0.056, respectively) with moderate performance levels, suggesting these factors might have less influence on online purchase intention (OPI) than previously assumed.

These IPMA results provide strategic direction for both practitioners and researchers, emphasizing the need to allocate resources towards enhancing information adoption (INA) and visual eWOM information credibility (INC) to potentially boost online purchase intention [142,143]. The analysis also indicates opportunities for a more balanced approach in managing different aspects of visual eWOM information, particularly by reallocating efforts from information usefulness (INU) to factors with greater impact.

**Fig 3:**
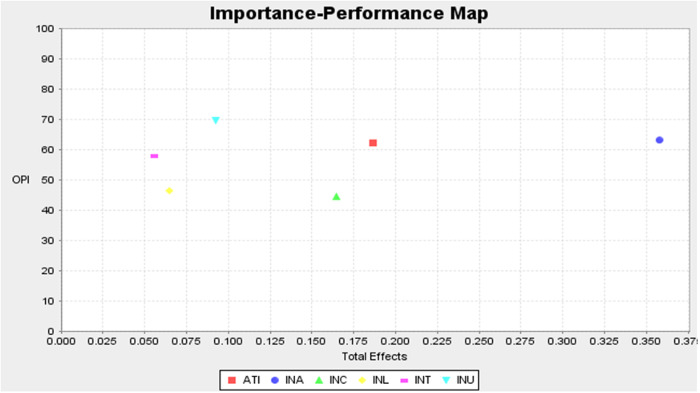
Importance and performance map (priority map) of IPMA for the customer’s online purchase intention.

Further exploration of the data through a priority map, presented in Fig 3, shows the performance levels on the vertical axis and the importance of factors on the horizontal gradient. The map reveals that information adoption (INA) and attitude toward information (ATI) are the most influential factors impacting online purchase intention. This indicates that customers are significantly affected by their acceptance of eWOM information and their overall attitude toward it when making online purchase decisions. These factors are crucial in shaping buying behavior in the digital marketplace. Conversely, factors such as information quantity (INT), information quality (INL), and information credibility (INC) should also be considered for a comprehensive strategy.

In conclusion, this study provides a clear understanding of the factors influencing online purchase intention through visual eWOM. The analysis confirms the reliability and validity of the constructs and measurements within this context. The research model demonstrates moderate explanatory power and predictive relevance, with information adoption (INA) and attitude towards information (ATI) emerging as critical drivers of consumer behavior. The findings underscore the vital role of information credibility (INC) in shaping consumer attitudes and adoption, highlighting the need for trustworthy visual eWOM content. While information usefulness (INU) performs well, its lower importance suggests a need for a more balanced focus across eWOM elements. The Importance-Performance Matrix Analysis (IPMA) identifies opportunities for improvement, particularly in enhancing the credibility and quality of visual eWOM content to better drive purchase intentions. Overall, the model provides valuable insights for optimizing digital marketing strategies by prioritizing credible, high-quality visual eWOM that aligns with consumer attitudes and promotes information adoption.

## 5. Discussion

This study underscores the pivotal role of visual eWOM characteristics in enhancing consumer engagement in online shopping, particularly through visual communication on social media platforms. The findings highlight several key relationships that align with existing theories and prior research, offering valuable insights into consumer behavior in the digital age.

The confirmed positive relationship between visual information quality (INL) and perceived information usefulness (INU) (H1) illustrates consumers’ strong preference for accurate, relevant, and up-to-date visual content. This aligns with the SOR theory proposed by Mehrabian and Russell [19], suggesting that high-quality visual stimuli enhance cognitive processing in consumers. This is also consistent with the findings of [14], which highlighted how high-quality visual content on social media platforms, especially brand-driven content, significantly influences consumer trust and engagement by providing relevant and accurate information. Moreover, the positive impact of INL on information adoption (INA) (H2) resonates with Jiang et al. [144], who found that consumers are more likely to adopt and trust information when it is perceived as high-quality and professionally presented. This suggests that high-quality visuals not only facilitate easier processing but also evoke positive emotions, encouraging quicker decision-making and fostering trust.

However, the absence of a direct effect between INL and attitude toward information (ATI) (H3) introduces complexity to the relationship. While high-quality visuals enhance comprehension and engagement, they do not independently shape attitudes. This suggests that other factors, such as source credibility and expertise, play crucial roles in attitude formation, as proposed by Cheung et al. [119]. This complexity in attitude formation is also observed in the study by Karpenka et al. [14], which indicated that while visual content is critical for trust-building in social media communities, it must be coupled with credible and authoritative sources to significantly impact consumer attitudes. This nuanced relationship between information quality and attitude formation warrants further investigation, especially in the context of visual eWOM.

The influence of visual information quantity (INT) in visual eWOM on consumer perceptions is complex, balancing both benefits and limitations. The study reveals that a higher quantity of visual eWOM can act as a heuristic cue, signaling comprehensiveness and positively impacting perceived information usefulness (INU) (H4). This finding aligns with Akdim [145], who emphasized that visual cues like videos can enhance the perceived effectiveness of reviews by making them easier to understand and more engaging. However, the lack of a significant relationship between information quantity (INT) and information adoption (INA) (H5), highlights the limitations of relying solely on the quantity of information to drive behavioral change. This outcome echoes the concept of information overload, where an excessive amount of visual stimuli can overwhelm consumers, triggering cognitive overload, a state of mental fatigue and confusion that impedes information processing and decision-making [146]. The findings by King et al. [147] further substantiate this notion, particularly within the context of visual eWOM, indicating that an abundance of visual stimuli, even if well-organized, can negatively affect user engagement. This suggests that the cognitive capacity of users to process visual information is limited, and exceeding this limit can lead to cognitive overload, hindering information adoption and behavioral change.

The concept of the mere exposure effect [148] plays a significant role in understanding how the quantity of visual eWOM influences consumer attitudes toward information (ATI) (H6). Repeated exposure to visual stimuli, such as multiple posts, shares, or visual comments, enhances content familiarity, leading to more favorable attitudes. This cognitive bias, where repeated exposure leads to a more favorable attitude, is supported by Nordhielm [149]. Yagi and Inoue [150], found that repeated exposure to advertising images increases brand awareness and positive attitudes. While the quantity of visual eWOM may not directly drive adoption, it establishes a foundation for future engagement by fostering favorable consumer attitudes. This underscores the importance for marketers to strike a balance in leveraging visual eWOM effectively, ensuring that the right elements capture consumer attention to maximize the benefits of the mere exposure effect.

The study highlights the significant influence of visual information credibility (INC) on information usefulness (INU) (H7), underscoring the crucial role credibility plays in enhancing the value and relevance of visual eWOM in consumer decision-making. This finding aligns with the research by Rofianto et al. [15], which revealed the importance of communicator credibility, specifically expertise and trustworthiness in shaping the credibility and usefulness of visual eWOM. Their study on YouTube “unboxing” videos demonstrates that higher credibility leads to increased adoption, emphasizing that when visual information originates from entities perceived as knowledgeable, reliable, and unbiased, it significantly enhances the information’s utility.

Beyond cognitive assessments of the usefulness of information, visual information credibility plays a crucial role in influencing consumer behavior, with a strong positive relationship observed between INC and information adoption (INA) (H8). When consumers perceive visual eWOM as credible, they are more likely to internalize and integrate the information into their existing beliefs and practices. This aligns with the concept of visual persuasion discussed by Joo et al. [151], where the persuasive power of images lies in their ability to communicate complex, non-verbal messages that shape audience perceptions and judgments. This is further reinforced by the idea that visual rhetoric not only conveys information but also validates and strengthens pre-existing beliefs and emotions, as noted by Kjeldsen [152]. In this context, source credibility becomes essential in determining whether consumers will adopt the information, much like how perceived blogger credibility influences message elaboration and brand attitude formation, as discussed by Chu and Kamal [153]. Therefore, the effectiveness of visual eWOM lies not only in its content but also in its ability to persuade and shape consumer behavior through credible and compelling imagery.

Moreover, INC exerts a significant influence on attitude toward information (ATI) (H9), with a strong association observed between visual information credibility and positive emotional responses, such as trust and confidence. This finding validates the SOR theory, indicating that credible stimuli evoke favorable emotional reactions, which, in turn, enhance consumer attitudes. The impact of eWOM varies across video platforms due to differences in content format and audience engagement. For instance, YouTube’s longer-form videos, which often feature detailed product reviews and tutorials, cater to viewers seeking in-depth information before making purchase decisions. This aligns with Djafarova and Rushworth [154], who found that longer videos on YouTube, when presented by credible sources, significantly influence consumer trust and purchase intention. The platform’s emphasis on expert opinions and comprehensive analysis makes eWOM particularly influential, especially for high-involvement or complex products that require detailed evaluations. In contrast, TikTok’s short-form videos prioritize entertainment, emotional engagement, and virality. These videos leverage trends and challenges to quickly capture attention and facilitate the rapid spread of eWOM. As highlighted by Filieri et al. [17], short, engaging videos are particularly effective in driving impulse purchases and promoting visually appealing products. TikTok’s algorithm-driven content discovery enhances eWOM dissemination, making it a powerful tool for influencing purchase behavior, particularly among younger demographics.

The confirmed positive relationship between INU and INA (H10) provides crucial insights into consumer engagement with visual eWOM. This finding suggests that perceived usefulness acts as a cognitive gateway, encouraging consumers to actively engage with and internalize the visual information presented. It aligns with previous research by Ngo et al. [155], which supports the idea that individuals are more likely to adopt information they find valuable and relevant to their decision-making processes. However, the absence of a direct effect between information usefulness INU and online purchase intention (OPI) (H11) reveals an intriguing nuance. While information usefulness is critical for information adoption, it alone does not drive purchase behavior. This aligns with the findings of Filieri et al. [17], which indicate that visual cues, such as user-generated pictures and performance heuristics, play a more substantial role in shaping consumers’ behavioral intentions and decisions. The effectiveness of visual eWOM is shaped by platform-specific narratives, where longer-form content on YouTube supports informed decision-making, while TikTok’s dynamic and trend-driven approach fosters immediate engagement and impulsive buying behavior. These differences highlight the need to consider platform-specific strategies when leveraging visual eWOM for marketing effectiveness. Despite the high quality of verbal information, such as detailed and accurate reviews, consumers are often more influenced by visual cues when forming purchase intentions. This suggests that while cognitive elements like information usefulness are foundational, they must be complemented by engaging visual content to effectively drive consumer actions. In the context of visual eWOM, this implies that while information usefulness might initiate cognitive processing and lead to information adoption, the subsequent affective responses ultimately propel consumers toward making a purchase. Visuals that evoke emotions such as excitement, trust, or aspiration can transform perceived usefulness into concrete actions. This finding underscores the importance of crafting visual eWOM that not only informs but also resonates with consumers on an emotional level.

The positive correlation between attitude toward information (ATI) and INA (H12) underscores the importance of consumer attitudes in processing information, particularly in digital environments. The study’s findings suggest that visual stimuli have a substantial impact on perceived cognition, indicating that when consumers hold a favorable attitude toward sensory-rich content, they are more likely to integrate this information into their decision-making processes. This finding is consistent with the research by Mardhatilah et al. [156], which explored the impact of audio-visual stimuli on consumer engagement in social media contexts. Their study demonstrated that sensory stimuli enhance the perceived cognitive appeal of content and play a crucial role in driving consumer engagement on platforms like Instagram. Therefore, fostering positive consumer attitudes toward content through strategic sensory marketing can significantly enhance information adoption and consumer engagement in digital settings.

Additionally, the significant positive relationship between ATI and OPI (H13) emphasizes the power of emotions in driving consumer behavior. A positive attitude, often fueled by the emotions elicited by visually appealing and engaging content, signals that the product or service is desirable and beneficial. This leads to a more positive evaluation of the offering and a stronger intention to purchase. This finding is consistent with the work of Lin et al. [12] who demonstrated that the inclusion of visual elements, such as pictures in blog posts, significantly enhances the perceived quality and credibility of eWOM messages, which, in turn, increases consumer interest and purchase intentions. Visual information helps consumers form emotional connections and better evaluate the product’s appeal, thereby strengthening their intention to purchase. By understanding the critical role of visual content in shaping consumer attitudes and purchase intentions, marketers can optimize their digital strategies by incorporating visually rich and emotionally engaging content, ultimately boosting consumer engagement and conversion rates.

Finally, this research confirms the significant positive influence of INA on OPI, establishing information adoption as a critical mediator between perceived visual eWOM characteristics and purchase intention. This finding not only aligns with prior research on eWOM information adoption [41] but also extends it significantly to the realm of visual eWOM, highlighting the necessity of active engagement and internalization for visual content to effectively translate into behavioral outcomes. Within the broader theoretical framework of the SOR model, information adoption functions as the critical organismic process that bridges the gap between stimuli (visual eWOM) and the behavioral response (online purchase intention). Our findings underscore the multifaceted nature of information adoption, which encompasses both cognitive and affective dimensions and acts as a conduit through which perceived visual eWOM characteristics influence purchase intention. When consumers encounter visual eWOM, they engage in a series of cognitive appraisals, evaluating the information’s credibility, relevance, quality, and usefulness. These appraisals determine the extent to which the information is perceived as valuable and trustworthy. If the cognitive appraisals are positive, consumers are more likely to adopt the information, integrating it into their mental models and knowledge structures. Furthermore, information adoption triggers affective responses, with credible, relevant, high-quality, and useful visual eWOM evoking positive emotions such as trust, excitement, or aspiration. These emotions reinforce cognitive evaluations and strengthen the consumer’s commitment to the information. This effective engagement further solidifies the mediating role of information adoption, bridging the gap between cognitive appraisal and behavioral intention. The dual nature of information adoption aligns with previous research emphasizing the importance of both cognitive and affective processes in explaining the impact of visual eWOM on consumer behavior [17]. Our study provides a nuanced understanding of the interplay between cognitive and affective factors in shaping consumer behavior in the context of visual eWOM, offering valuable insights for marketers aiming to optimize the persuasive impact of visual content in digital environments.

### 5.1. Implications

#### 5.1.1. Theoretical implications.

This research makes several key theoretical contributions to the understanding of visual eWOM and its role in shaping consumer behavior. First, it extends the SOR model to the context of visual eWOM on social media. While prior research has primarily focused on textual eWOM, this study emphasizes how visual elements serve as stimuli that trigger cognitive and affective processes, ultimately influencing purchase intention. By integrating visual communication into the SOR framework, this study provides a more comprehensive understanding of consumer decision-making in a digital environment where visual content is increasingly dominant.

Second, this research introduces information adoption (INA) as a critical mediator in the relationship between visual eWOM characteristics and purchase intention. Our findings demonstrate that while information quality, quantity, and credibility influence perceived usefulness and attitudes, it is the active adoption of the information – the process of internalizing and integrating it into one’s existing knowledge and beliefs – that ultimately drives purchase behavior. This finding significantly extends previous eWOM research, which has often focused on direct relationships between eWOM characteristics and purchase intention, without fully considering the crucial role of information adoption as a necessary intervening step. This insight adds a new dimension to eWOM literature by demonstrating that information adoption is not just an outcome but a pivotal mechanism in visual communication’s effectiveness.

Third, the study reveals a nuanced interplay between cognitive and affective processes. Unlike textual eWOM, where information quantity plays a more direct role, our findings show that credibility is the strongest predictor of attitude, underscoring the importance of trustworthiness in visual contexts. Additionally, while information quality enhances perceived usefulness (a cognitive evaluation), it does not directly influence attitude toward information. This suggests that in the visual domain, the believability of the source is more influential in shaping consumers’ emotional responses than the sheer volume of information presented. Moreover, the study highlights the risk of information overload in visually rich environments, where excess information may not necessarily lead to adoption, setting it apart from textual eWOM studies.

Finally, the study challenges the assumption that information usefulness alone can drive purchase intention. While perceived usefulness is a prerequisite for information adoption, it must be coupled with a positive attitude and the active integration of information to effectively influence purchasing decisions. This finding contributes to a more holistic and sequential understanding of consumer decision-making in the context of visual eWOM. By revealing how cognitive and affective responses interact in a visually driven environment, this study advances the theoretical discourse on digital consumer behavior and offers valuable insights for future research on social media marketing.

#### 5.1.2. Managerial implications.

This study provides actionable insights for marketers aiming to leverage visual eWOM on social media effectively. To maximize the impact of visual eWOM on consumer behavior, brands should focus on four key areas: content quality, credibility, engagement strategies, and performance measurement.

The demonstrated significance of information quality on both information usefulness and adoption underscores the critical need for high-quality visuals. This extends beyond aesthetics; brands should invest not only in professional photography and videography but also in ensuring the accuracy, relevance, and clarity of visual content. Visuals should directly address consumer needs and accurately represent product features. While a visually engaging presence is important (supported by the positive correlation between information quantity and attitude), marketers must balance quantity with quality to avoid cognitive overload. Regularly posting aesthetically pleasing content is a starting point, but encouraging user-generated visual content (UGVC) is crucial for building authenticity and social proof. Visual storytelling, showcasing real customer experiences, can foster emotional connections and enhance credibility.

The finding emphasizes the critical role of information credibility in influencing consumer behavior. To establish trust and authenticity, marketers should prioritize transparency and authenticity in all visual eWOM communications. This means partnering with credible influencers whose values align with the brand, clearly disclosing sponsored content, and ensuring visual representations of products or services are accurate and not misleading. Furthermore, actively managing visual eWOM is essential. This involves promptly responding to customer reviews and comments (both positive and negative), addressing concerns publicly and transparently, and actively promoting positive UGVC. A/B testing different visual styles and messaging can help optimize content for engagement and adoption.

Finally, understanding the crucial role of information adoption means marketers should design visual campaigns that actively encourage interaction and internalization. This could involve incorporating interactive elements like polls, quizzes, or questions within visual content, prompting users to share their own experiences, or using calls to action that encourage consumers to learn more or try the product. Ultimately, a successful visual eWOM strategy requires a holistic approach that prioritizes quality, credibility, engagement, and a deep understanding of how consumers process and adopt visual information. Measuring the performance of these efforts by examining information adoption metrices are crucial.

### 5.2. Limitations and future research directions

While this study provides meaningful insights into the impact of visual eWOM on purchase intention, several limitations should be acknowledged. First, the cross-sectional design of the study restricts the ability to establish definitive causal relationships between the variables. Although the SOR model offers a strong theoretical framework, a longitudinal approach would allow for a better understanding of the temporal dynamics of visual eWOM and its sustained impact on consumer behavior. Future research should explore how these relationships evolve over time, particularly as consumers are exposed to repeated visual eWOM interactions. Second, this study did not focus on a specific social media platform. Different platforms (e.g., Instagram, TikTok, YouTube) have distinct visual aesthetics, content formats, and user engagement patterns, which may moderate the observed relationships. Future research should conduct comparative studies across platforms to identify whether and how the effectiveness of visual eWOM varies depending on platform-specific features and audience characteristics. Third, while this study acknowledges that visual eWOM can be manipulated or misleading, it does not explore how consumers perceive and react to deceptive content. Future research should examine consumer responses to deceptive or manipulated visual eWOM and investigate strategies brands can use to maintain trust and credibility. Finally, the use of convenience sampling may limit the generalizability of the findings to broader consumer populations and cultural contexts. Future research could address this by employing probability sampling techniques and exploring cross-cultural variations in consumer responses to visual eWOM.

## 6. Conclusion

This study offers a comprehensive examination of the relationship between visual electronic word-of-mouth (eWOM) and online purchase intention, within the framework of the SOR theory. By employing a quantitative approach with a sample of 335 participants and utilizing Partial Least Squares Structural Equation Modeling (PLS-SEM), the research provides nuanced insights into how different aspects of visual eWOM specifically information quality, quantity, and credibility shape consumer perceptions, information adoption, and attitudes. The findings underscore the pivotal role of information adoption as a mediator in the relationship between visual eWOM and purchase intention. This underscores the importance of active engagement and internalization of visual eWOM content for influencing consumer behavior. This process involves more than just passive exposure to visual content; it requires active engagement, cognitive processing, and the integration of information into consumers’ belief systems. Notably, the study reveals that increasing the quantity of visual eWOM content does not necessarily lead to higher information adoption, challenging the assumption that more content automatically drives behavioral change. Additionally, the lack of a direct relationship between information usefulness and online purchase intention suggests that usefulness alone is insufficient to influence consumer decisions.

The study’s theoretical contributions lie in extending the SOR framework into the realm of visual eWOM and elucidating the dynamic process of information adoption as a central mechanism through which visual stimuli influence consumer behavior. From a managerial perspective, the findings offer actionable insights for marketers seeking to leverage the persuasive power of visual communication on social media platforms. By prioritizing high-quality, credible, and engaging visuals, marketers can enhance the perceived usefulness of their messages, foster positive consumer attitudes, and ultimately drive purchase intention. However, the study’s cross-sectional design and reliance on convenience sampling limit the generalizability of the findings. Future research could address these limitations by employing longitudinal designs and probability sampling to explore the long-term effects of visual eWOM and ensure broader applicability of the results. Despite these limitations, this study contributes valuable knowledge to the field of visual eWOM and its impact on consumer behavior, offering both theoretical advancements and practical guidance for marketing strategies.

## Supporting information

S1 File Survey questionnaire. (PDF)

S2 File Dataset used for analysis. (XLSX)
