# Supplementary material for: How perceived eWOM in visual form influences online purchase intention on social media: A research based on the SOR theory
Source: PLoS One. 2025 Jul 10;20(7):e0328093. doi: 10.1371/journal.pone.0328093 (PMC12244485; doi:10.1371/journal.pone.0328093)
Supplement: S1 File — (PDF) [file pone.0328093.s001.pdf]

QUESTIONNAIRE

How Perceived eWOM in Visual Form Influences Online Purchase Intention on Social Media. A Research Based on The SOR Theory

Dear Participants,

We are conducting a study to explore how visual electronic word-of-mouth (visual eWOM) shared on social media influences consumer decisions and online purchase intentions. Specifically, we aim to understand how factors such as information credibility, quantity, usefulness, adoption, and attitudes toward the information you encounter online affect your buying behavior.

Your participation is entirely voluntary and anonymous, and all responses will be kept confidential and used solely for research purposes. We sincerely appreciate your time and effort in completing this survey.

Thank you for your support!

I. Do you consent to participate in this study and allow the information you provide to be used for research purposes?

☐ Yes
☐ No

II. Demographics

1. Gender:

☐ Male
☐ Female
☐ Other

2. Age:

☐ 18-21
☐ 22-25
☐ Over 25

III. Visual eWOM Activity

Visual eWOM (electronic word-of-mouth) refers to consumer-generated visual content – such as images, videos, infographics, or product demonstrations – shared across online platforms to express opinions or experiences about products, services, or brands (Li et al., 2024).

1. **Do you participate in Visual eWOM activities on social networking sites?**

☐Yes
☐ No

2. **Social networking sites usage and Visual eWOM behavior**

2.1 Which social networking sites do you use the most?

☐ Facebook
☐ Instagram
☐ TikTok
☐ YouTube
☐ Other: __________

2.2 Daily time spent on social networking sites:

☐ Less than 1 hour
☐ From 1 hour to 2 hours
☐ From 2 hours to 3 hours
☐ More than 3 hours

IV. Factors Influencing consumer purchase intention toward Visual

The following section measures respondent perceptions on Visual eWOM information and its influence on purchase intention. Please use a five-point Likert scale

1 - Strongly Disagree 2 - Disagree 3 - Neutral 4 - Agree 5 - Strongly Agree

**Information Quality**	1	2	3	4	5
1) I can understand visual eWOM information shared in social media.					
2) I think visual eWOM information shared in social media is clear.					
3) I find visual eWOM information in social media relevant to my needs.					
4) I think visual eWOM information in social media is detailed.					
**Information Quantity**					
1) I can rely on the amount of visual eWOM information in social media.					

2) The amount of visual eWOM information in social media can help me understand the product performance.					
**Information Credibility**					
1) I think visual eWOM information in social media is convincing.					
2) I think visual eWOM information in social media is credible.					
3) I think visual eWOM information in social media is believable.					
4) I think visual eWOM information in social media is trustworthy.					
**Information Usefulness**					
1) Visual eWOM information in social media is generally useful for me to evaluate the product.					
2) Visual eWOM information in social media is generally helpful for me to evaluate the product.					
3) Visual eWOM information in social media is generally informative for me to evaluate the product.					
**Information Adoption**					
1) I learn something new of brands through visual eWOM information in social media.					
2) I accept the visual eWOM information of brands in social media.					
3) I accept the visual eWOM recommendation of brands in social media.					
**Attitude towards Information**					
1) I always review visual eWOM information in social media before buying a product.					
2) I find visual eWOM information in social media helpful for making purchase decisions.					
3) I feel visual eWOM information in social media makes me more confident in purchasing a product.					

**Online Purchase Intention**					
1) It is very likely that I will buy the product.					
2) I will buy the product next time I need a product.					
3) I will recommend the product to my friend.					

Thank you once again for your support!

Research Team
